# Added Value of Next Generation over Sanger Sequencing in Kenyan Youth with Extensive HIV-1 Drug Resistance

**DOI:** 10.1128/spectrum.03454-22

**Published:** 2022-11-29

**Authors:** V. Novitsky, W. Nyandiko, R. Vreeman, A. K. DeLong, A. Manne, M. Scanlon, A. Ngeresa, J. Aluoch, F. Sang, C. Ashimosi, E. Jepkemboi, M. Orido, J. W. Hogan, R. Kantor, for the RESistance in a PEdiatric CohorT (RESPECT) Study

**Affiliations:** a Brown University, Providence, Rhode Island, USA; b Academic Model Providing Access to Healthcare (AMPATH), Eldoret, Kenya; c Moi University, Eldoret, Kenya; d Icahn School of Medicine at Mount Sinai, New York, New York, USA; e Arnhold Institute for Global Health, New York, New York, USA; Karolinska Institutet

**Keywords:** drug resistance testing, HIV-1, Sanger sequencing, next-generation sequencing

## Abstract

HIV-1 drug resistance testing in children and adolescents in low-resource settings is both important and challenging. New (more sensitive) drug resistance testing technologies may improve clinical care, but evaluation of their added value is limited. We assessed the potential added value of using next-generation sequencing (NGS) over Sanger sequencing for detecting nucleoside reverse transcriptase inhibitor (NRTI) and nonnucleoside reverse transcriptase inhibitor (NNRTI) drug resistance mutations (DRMs). Participants included 132 treatment-experienced Kenyan children and adolescents with diverse HIV-1 subtypes and with already high levels of drug resistance detected by Sanger sequencing. We examined overall and DRM-specific resistance and its predicted impact on antiretroviral therapy and evaluated the discrepancy between Sanger sequencing and six NGS thresholds (1%, 2%, 5%, 10%, 15%, and 20%). Depending on the NGS threshold, agreement between the two technologies was 62% to 88% for any DRM, 83% to 92% for NRTI DRMs, and 73% to 94% for NNRTI DRMs, with more DRMs detected at low NGS thresholds. NGS identified 96% to 100% of DRMs detected by Sanger sequencing, while Sanger identified 83% to 99% of DRMs detected by NGS. Higher discrepancy between technologies was associated with higher DRM prevalence. Even in this resistance-saturated cohort, 12% of participants had higher, potentially clinically relevant predicted resistance detected only by NGS. These findings, in a young, vulnerable Kenyan population with diverse HIV-1 subtypes and already high resistance levels, suggest potential benefits of more sensitive NGS over existing technology. Good agreement between technologies at high NGS thresholds supports their interchangeable use; however, the significance of DRMs identified at lower thresholds to patient care should be explored further.

**IMPORTANCE** HIV-1 drug resistance in children and adolescents remains a significant problem in countries facing the highest burden of the HIV epidemic. Surveillance of HIV-1 drug resistance in children and adolescents is an important public health strategy, particularly in resource-limited settings, and yet, it is limited due mostly to cost and infrastructure constraints. Whether newer and more sensitive next-generation sequencing (NGS) adds substantial value beyond traditional Sanger sequencing in detecting HIV-1 drug resistance in real life settings remains an open and debatable question. In this paper, we attempt to address this issue by performing a comprehensive comparison of drug resistance identified by Sanger sequencing and six NGS thresholds. We conducted this study in a well-characterized, vulnerable cohort of children and adolescents living with diverse HIV-1 subtypes in Kenya and, importantly, failing antiretroviral therapy (ART) with already extensive drug resistance. Our findings suggest a potential added value of NGS over Sanger even in this unique cohort.

## INTRODUCTION

The impact of HIV-1 drug resistance on antiretroviral treatment (ART) options and outcomes in children and adolescents remains a significant problem in African countries (1–3). Limited treatment options and the high cost of advanced regimens are major challenges to ART, particularly in children and adolescents, who face treatment barriers, including high viral loads, a wide treatment gap between those who need ART and those receiving it, low rates of virologic suppression, limited ART formulations, and substantial nonadherence (2, 4, 5).

HIV-1 drug resistance testing is important for choosing optimal ART regimens, preventing treatment failure, detecting drug resistance evolution, and guiding lifelong ART. For decades, chain-terminating dideoxynucleotide population Sanger sequencing has been used for HIV drug resistance testing. Major limitations of Sanger sequencing are cost and particularly its inability to detect minority resistance variants below ~20% of the viral quasispecies (6–10). These minority variants could be important in further resistance development (11–17), including in children (18–22). While next-generation sequencing (NGS) can detect drug resistance mutations at better sensitivity than Sanger (17, 23–25) and some NGS-based drug resistance testing platforms are already validated and commercially available, its advantages over Sanger sequencing remain uncertain. If proven beneficial, this evolving technology could become a more recommended platform for HIV drug resistance testing for research, routine clinical care, and surveillance (23, 24, 26).

Agreements and discrepancies between Sanger sequencing and NGS in the identification of HIV-associated drug resistance mutations (DRMs) were assessed in previous studies using a variety of NGS protocols and platforms (12, 19, 23, 26–41). Most studies found good agreement between these technologies in identifying DRMs at high (>20%) detection thresholds, while as expected, NGS was superior at lower (<20%) thresholds. However, most studies were relatively small; targeted mostly adults; included predominantly HIV-1 subtype B sequences from developed countries; analyzed few selected NGS thresholds; or included treatment-naive individuals, “artificial” laboratory specimens, or samples with limited clinical characterization or with low levels of drug resistance. Little is known about agreement between Sanger and NGS in real-life data sets, particularly from children and adolescents living with diverse HIV-1 subtypes in low-resource settings and failing ART. It remains unclear whether NGS could provide any added value over Sanger sequencing for identifying potentially clinically relevant DRMs in such settings and vulnerable populations.

In this study, we conducted both Sanger sequencing and NGS at the same time point in a longitudinally followed cohort of children and adolescents living with HIV in Kenya, and we characterized agreements and discrepancies in drug resistance detection between these technologies. We hypothesized that the higher sensitivity of NGS over Sanger sequencing to detect DRMs could improve the estimation of drug resistance levels to current and future treatment options and potentially improve patient care even in this cohort, which already has high levels of drug resistance.

## RESULTS

### Study cohort.

A total of 132 virologically unsuppressed children and adolescents had Sanger and NGS sequences and were included in the analyses. Demographic, clinical, and HIV-1 subtyping data for participants are presented in Table 1; a total of 48% were female, with a median age of 8.4 years (interquartile range [IQR], 6.1 to 10.5; range, 1.5 to 14.9) and a median time on ART at enrollment of 2.7 years (IQR, 1.5 to 4.1; range, 0.1 to 7.7). All participants were on nucleoside reverse transcriptase inhibitor (NRTI)- and nonnucleoside reverse transcriptase inhibitor (NNRTI)-containing regimens. The median CD4 count among study participants was 591 cells/μL (IQR, 240 to 949), and the median CD4 percentage was 22 (IQR, 13 to 31). The median viral load was 3.9 log_10_ copies/mL (IQR, 3.5 to 4.4). The majority of study participants (99/132, 75%) had HIV-1 subtype A1, 21 (16%) had subtype D, 4 (3%) had subtype C, and 2 had A/D and 2 had C/D recombinant viruses.

**TABLE 1:** Demographic, clinical, and HIV-1 subtyping data of the study cohort^*a*^

Parameter	Value
Sex (*n* [%])	
Males	68 (52)
Females	64 (48)
Age (median yrs [IQR])	8.4 (6.1–10.5)
Time on ART (median yrs [IQR])	2.7 (1.5–4.1)
Treatment regimens (*n* [%])	
3TC, ABC, NVP	62 (47)
3TC, ABC, EFV	15 (11)
3TC, AZT/D4T, NVP	48 (36)
3TC, AZT/D4T, EFV	7 (5)
CD4 count, cells/μL (median [IQR])	591 (240–949)
CD4 percentage (median [IQR])	22 (13–31)
HIV-1 RNA load, copies/mL (median [IQR])	7,660 (2,850–24,120)
HIV-1 subtypes (*n* [%])	
HIV-1 subtype A1	99 (75)
HIV-1 subtype D	21 (16)
HIV-1 subtype C	4 (3)
Other HIV-1 subtypes and recombinants	8 (6)

a N = 132. 3TC, lamivudine; ABC, abacavir; ART, antiretroviral therapy; AZT, zidovudine; D4T, stavudine; EFV, efavirenz; IQR, interquartile range; NVP, nevirapine.

The extent of NRTI and NNRTI drug resistance by Sanger sequencing in the cohort was high. Any DRMs were present in 94% of participants, NRTI DRMs in 89%, NNRTI DRMs in 94%, and dual-class resistance in 89%. The median number of NRTI+NNRTI DRMs per participant was 5 (IQR, 3 to 6), that of NNRTI was 2 (IQR, 1 to 3; range, 0 to 6), and that of NRTI was 3 (IQR, 1 to 4; range 0 to 8). These high resistance levels were consistent with our recent (nonidentical) report from this cohort, where 93% of youth had any DRMs (93% NRTI, 93% NNRTI, and 89% dual class) (42).

### Sanger versus NGS agreement in drug resistance detection.

**FIG 1:**
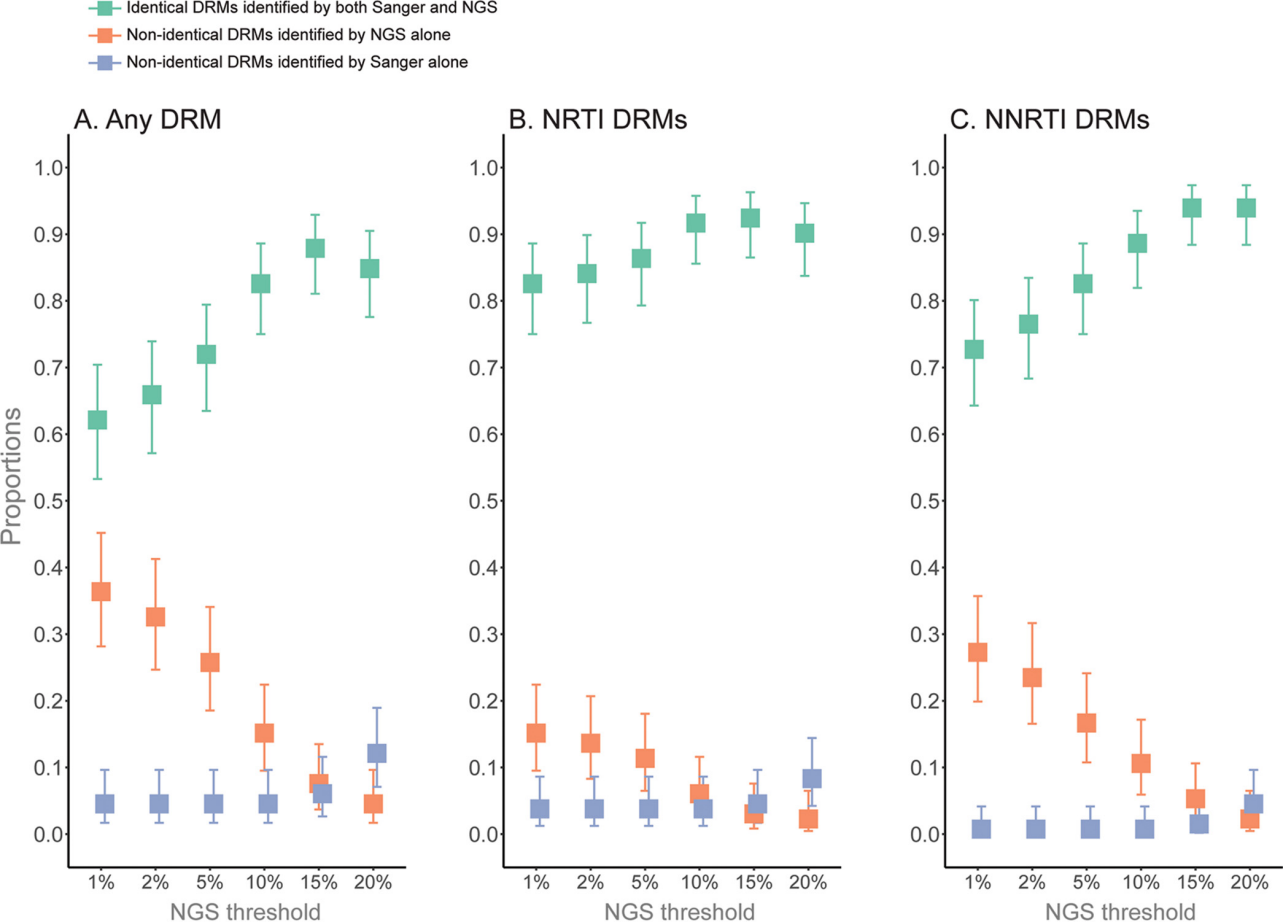
Agreement and disagreement between DRMs identified by Sanger and NGS. The figure presents proportions (*y* axes) of any DRMs (A), NRTI DRMs (B), and NNRTI DRMs (C) that were detected for each participant by Sanger and the six examined NGS thresholds (*x* axes). Identical DRM profiles identified by both sequencing technologies were considered an agreement. Proportions of individuals with identical and not identical profiles are represented by the colored squares according to the legend. A single binary outcome was assigned to every participant for each NGS threshold. Error bars show lower and higher 95% confidence intervals.

In the first analysis, depending on the NGS threshold, 62% to 88% of participants had full agreement of all their resistance profiles between Sanger and NGS (NRTI and/or NNRTI), 83% to 92% for NRTI DRMs, and 73% to 94% for NNRTI DRMs (Fig. 1). The proportion of participants with DRMs identified by NGS alone reduced gradually with increasing NGS thresholds, as follows: any DRM from 36% at NGS^01^ to 5% at NGS^20^, NRTI DRMs from 15% at NGS^01^ to 2% at NGS^20^, and NNRTI DRMs from 27% at NGS^01^ to 2% at NGS^20^. (Note that for clarity and distinction from the DRM prevalence rates, NGS thresholds are presented as superscripts throughout the text; e.g., NGS^01^ indicates NGS threshold at 1%, NGS^02^ for 2%, etc.). DRMs were identified by Sanger alone at high NGS thresholds. For example, 12% of any DRM, 8% of NRTI DRMs, and 5% of NNRTI DRMs were detected by Sanger but not by NGS^20^.

In the second analysis, we identified overall 635 DRMs by Sanger and 619 mutations at NGS^20^, 635 at NGS^15^, 654 at NGS^10^, 676 at NGS^05^, 697 at NGS^02^, and 715 at NGS^01^ thresholds. The mean number of DRMs per participant identified by both Sanger and NGS was 4.6 to 4.7 for total NRTI+NNRTI DRMs, 2.6 to 2.7 for NRTI DRMs, and 2.1 for NNRTI DRMs (Table 2). Depending on the threshold, NGS identified 96% to 100% of DRMs detected by Sanger sequencing, while Sanger identified 83% to 99% of DRMs detected by NGS. Overall, NNRTI minority DRMs detected by NGS and not by Sanger were more frequent than NRTI minority DRMs (18% versus 9%, respectively; *P* < 0.001, Fisher’s exact test). The mean number of DRMs identified by NGS alone decreased gradually across increasing NGS thresholds, while the mean number of DRMs identified by Sanger sequencing alone was relatively stable.

**TABLE 2:** Mean number of DRMs per study participant identified by Sanger sequencing and NGS at different detection thresholds^*a*^

ARV class	NGS detection threshold (%)	Mean no. of DRMs by:		
		Agreement (Sanger and NGS)	Discrepancy	
			NGS alone	Sanger alone
Any DRM (NRTI and/or NNRTI)	1	4.65	0.67	0.07
	2	4.65	0.54	0.07
	5	4.65	0.38	0.07
	10	4.65	0.21	0.07
	15	4.63	0.09	0.09
	20	4.55	0.05	0.17
NRTI DRMs	1	2.65	0.24	0.06
	2	2.65	0.20	0.06
	5	2.65	0.16	0.06
	10	2.65	0.08	0.06
	15	2.64	0.04	0.08
	20	2.59	0.02	0.12
NNRTI DRMs	1	2.09	0.43	0.01
	2	2.09	0.33	0.01
	5	2.09	0.22	0.01
	10	2.09	0.13	0.01
	15	2.08	0.05	0.02

a ARV, antiretroviral; DRMs, drug resistant mutations; NGS, next generation sequencing; NRTI, nucleoside reverse transcriptase inhibitors; NNRTI, nonnucleoside reverse transcriptase inhibitors.

**FIG 2:**
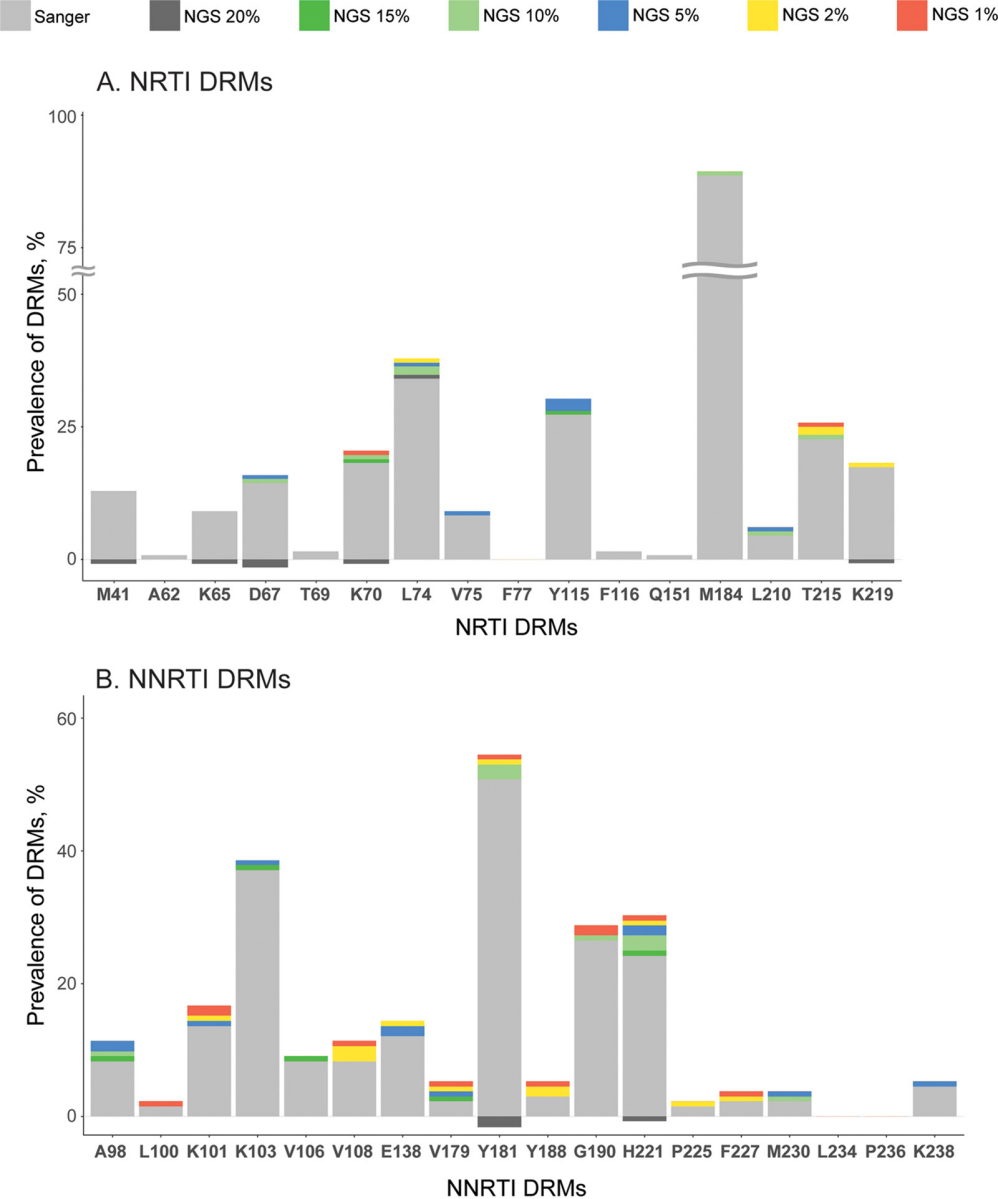
Prevalence of NRTI and NNRTI DRMs identified by Sanger and NGS. The stacked bar figure presents prevalence (*y* axes) of specific NRTI (A) and NNRTI (B) DRMs that were detected in this study, according to which sequencing technology and NGS threshold they were detected by, as indicated in the legend. Negative values indicate cases identified by Sanger alone.

Overall, NGS identified more DRMs than Sanger, at all thresholds (Fig. 2, NGS colored bars on top of the Sanger gray bars). In contrast, few DRMs were identified by Sanger alone (Fig. 2, negative-value bars), which was attributable mostly to NGS^20^. This pattern was observed for both NRTI- (Fig. 2A) and NNRTI-associated (Fig. 2B) DRMs. The most common DRMs were NRTI M184V and NNRTI Y181C.

**FIG 3:**
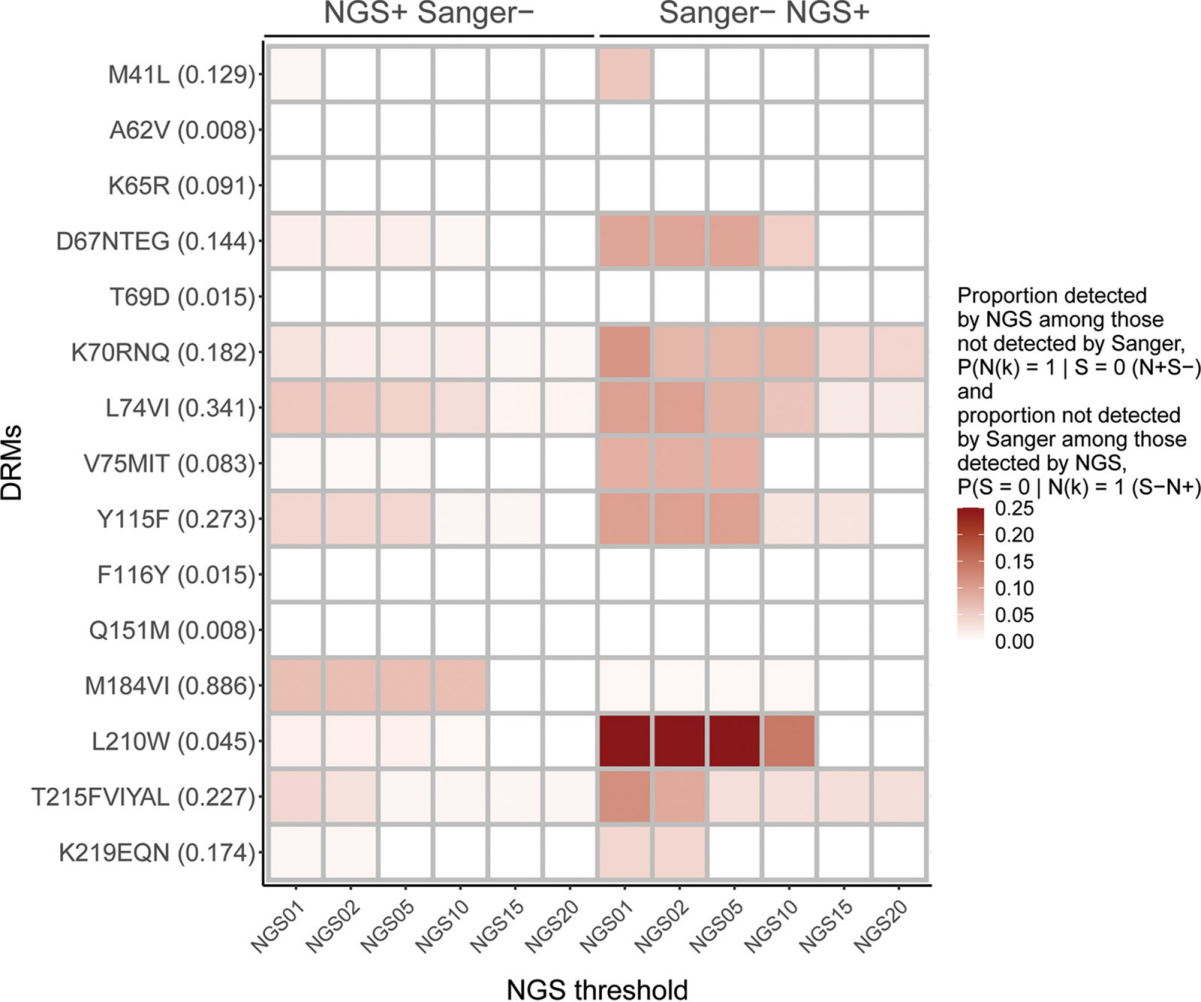
Discrepancy in detecting specific NRTI DRMs. The heatmap shows specific NRTI DRMs and their proportions (in parenthesis; *y* axis) according to Sanger sequencing and each of the six NGS thresholds (*x* axis). The left half of the heatmap indicates DRMs that were detected by NGS among those not detected by Sanger, shown as “NGS+ Sanger–.” The right half of the heatmap indicates DRMs that were not detected by Sanger among those detected by NGS, shown as “Sanger– NGS+.” The intensity of the heatmap colors ranges from 0 to 0.25 according to the scale at the right of the graph. See also Table S3 in the supplemental material.

**FIG 4:**
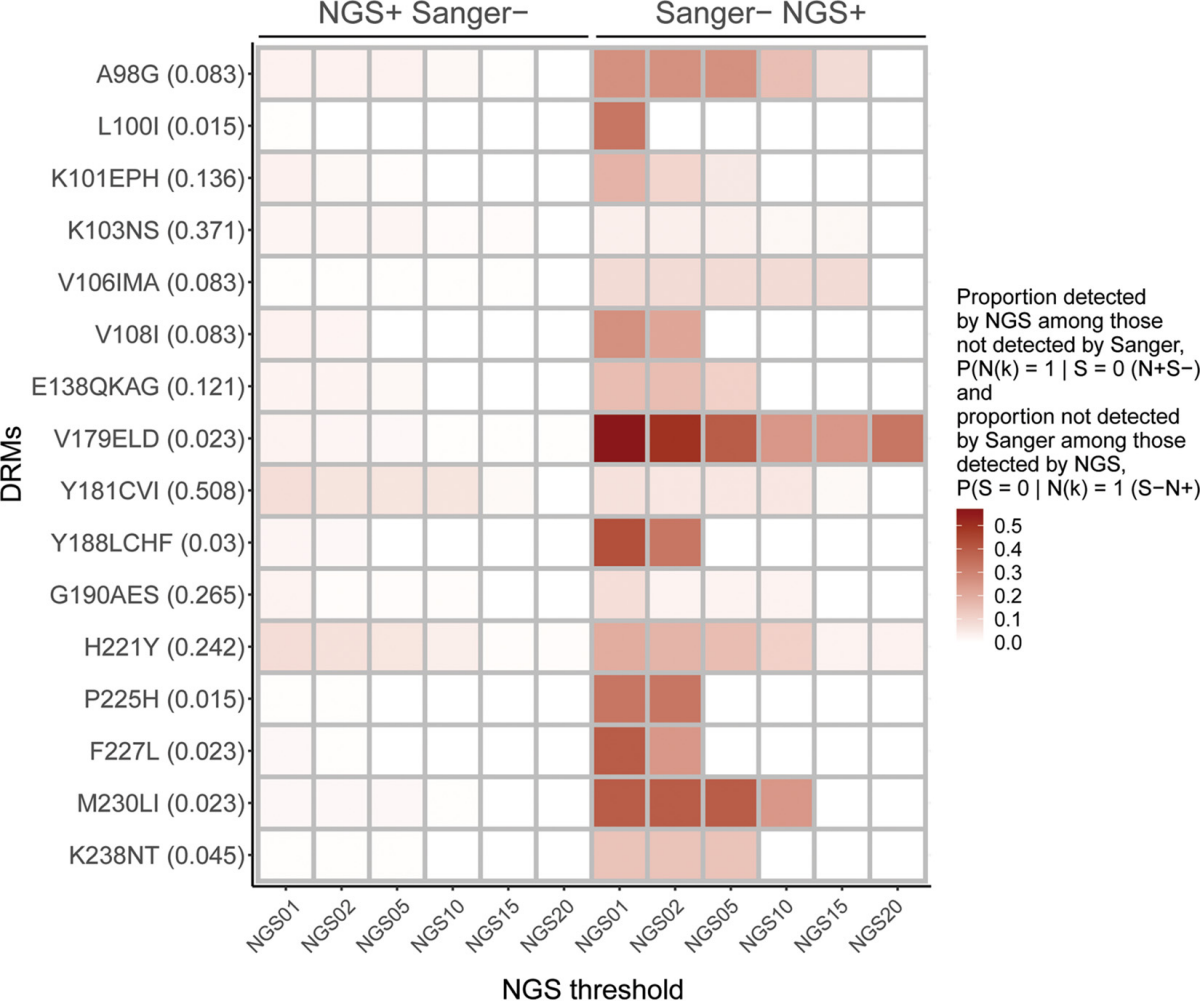
Discrepancy in detecting specific NNRTI DRMs. The heatmap shows specific NNRTI DRMs and their proportions (in parenthesis; *y* axis) according to Sanger sequencing and each of the six NGS thresholds (*x* axis). The left half of the heatmap indicates DRMs that were detected by NGS among those not detected by Sanger, shown as “NGS+ Sanger–.” The right half of the heatmap indicates DRMs that were not detected by Sanger among those detected by NGS, shown as “Sanger– NGS+.” The intensity of the heatmap colors ranges from 0 to 0.50 according to the scale at the right of the graph. See also Table S3.

For the third analysis, Fig. 3 and 4 illustrate discordance in the detection of specific NRTI and NNRTI DRMs. The left side of each figure shows the DRMs (and their proportions) that were detected by different NGS thresholds and not by Sanger. The right side of each Figure shows the DRMs (and their proportions) that were not detected by Sanger but were detected by the different NGS thresholds (treating NGS as a gold standard, this would be the false-negative rate associated with using Sanger to detect DRMs). Five of the 15 NRTI DRMs (Fig. 3) and none of the 16 NNRTI DRMs (Fig. 4) were concordant across all NGS thresholds. In general, both measures of discordance improved at the higher thresholds.

**FIG 5:**
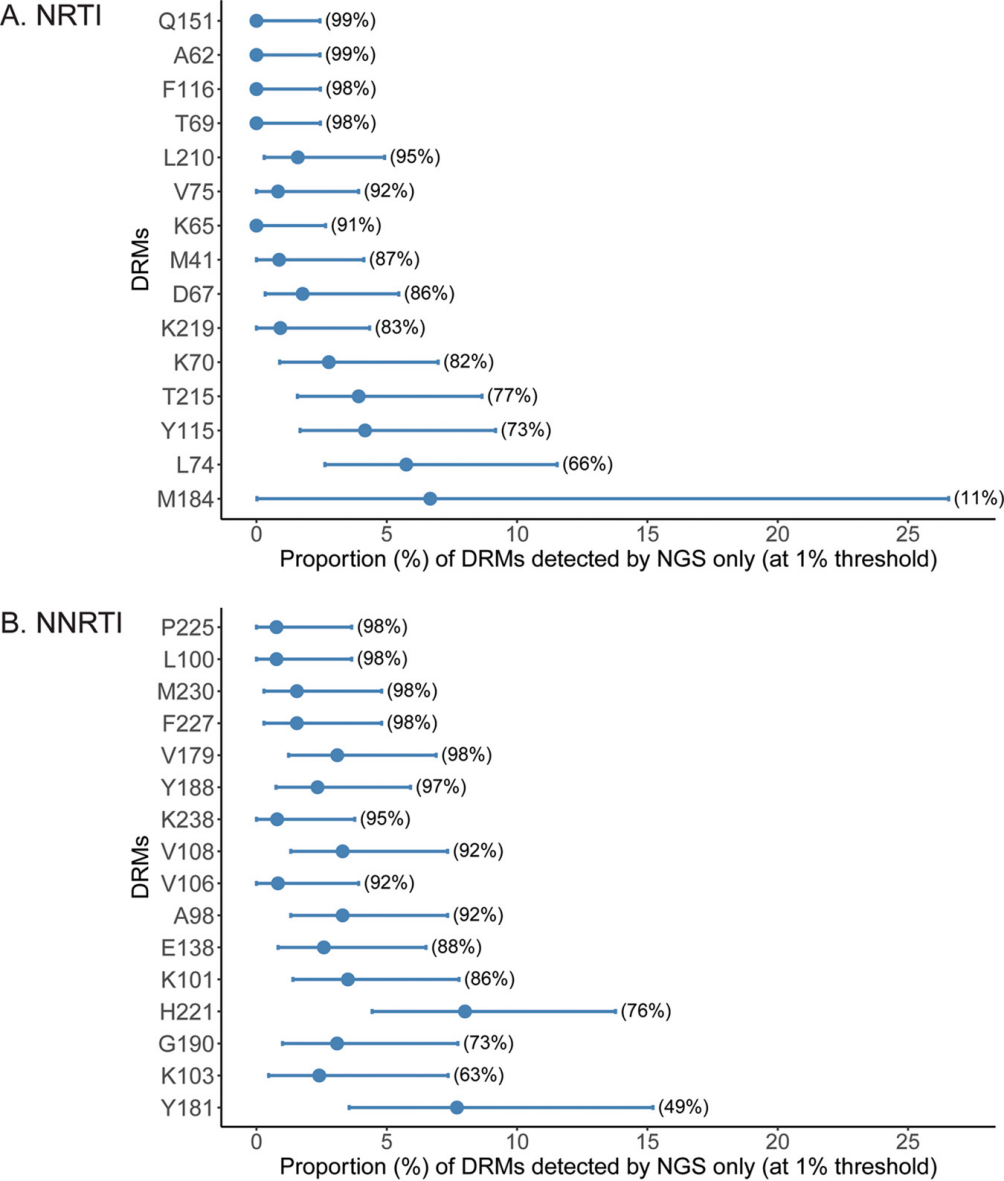
Discrepancy in the detection of specific DRMs. The figure presents estimates and associated Agresti-Coull 95% confidence intervals of the proportion (*x* axis) that each NRTI (A) and NNRTI (B) DRM (*y* axis) was detected by NGS at the 1% threshold, among the Sanger genotypes that did not detect that DRM. The proportion of Sanger genotypes where the DRM was not detected is provided in parentheses. Mutations without a vertical lower bound visible have lower bound coinciding with the estimate of 0. The figure is sorted by the proportion in which the mutation was detected by Sanger (see Fig. 3 and 4).

Figure 5 shows the proportion (and associated 95% CI) of each DRM that was detected by NGS at the 1% threshold among the Sanger genotypes in which this DRM was not detected. For the NRTIs, M184 and L74 had the highest rates of discordance (6.7% and 5.7%), and for the NNRTIs, Y181 and H221 had the highest discordance (7.7% and 8.0%).

The likelihood ratio test of the hypothesis that this type of discordance differs across DRMs did not show evidence of such variation (*P* = 0.28 for NRTI and *P* = 0.08 for NNRTI); however, in a small sample, these tests may be underpowered.

Spearman’s rank correlation was 0.90 for NRTI and 0.70 for NNRTI, indicating that DRMs with higher prevalence by Sanger had higher discordance rates.

### Sanger versus NGS agreement in predicting drug resistance.

In the fourth analysis to evaluate predicted drug resistance, 123/132 (93%) participants had Sanger- as well as NGS-predicted low-intermediate-high drug resistance to at least one drug in their regimen, ranging from 65% for D4T to 96% for NVP (Table 3). However, even in this resistance-saturated cohort, five additional participants had predicted resistance detected only by NGS, for lamivudine (3TC) (NGS thresholds, 1% to 10%), ABC (1% to 20%), zidovudine (AZT) (1% to 15%), efavirenz (EFV) (1% to 15%), and nevirapine (NVP) (1% to 15%). One participant with AZT predicted resistance was identified by Sanger and missed by NGS^20^, but it was detected at all other NGS thresholds.

**TABLE 3:** Participants with predicted low-intermediate-high drug resistance to antiretroviral drugs in current regimens

Resistance predicted by:	Results (*n* [%])^*a*^ by ARV drug					
	3TC (*n* = 132)	ABC (*n* = 77)	D4T (*n* = 23)	AZT (*n* = 32)	EFV (*n* = 22)	NVP (*n* = 110)
NGS and Sanger	117 (89)	64 (83)	15 (65)	22^*b*^ (69)	17 (77)	106 (96)
NGS only	1^*c*^ (1)	1 (1)	0 (0)	1 (3)	1^*c*^ (5)	1^*b*^ (1)
Sanger only	0 (0)	0 (0)	0 (0)	1^*d*^ (3)	0 (0)	0 (0)

a The denominator for all proportions was the number of study participants with the specific drug in their current regimen. NGS, next generation sequencing; 3TC, lamivudine; ABC, abacavir; D4T, stavudine; AZT, zidovudine; EFV, efavirenz; NVP, nevirapine.

b For one participant, at all thresholds except NGS^20^.

c For one participant, at all thresholds except NGS^15^ and NGS^20^.

d For one participant, at all thresholds except NGS^20^.

In the same analysis, 110/132 (83%) participants had Sanger- as well as NGS-predicted low-intermediate-high drug resistance to at least one future drug, ranging from 32% (tenofovir [TDF]) to 80% (rilpivirine [RPV]) (Table 4). Eleven additional participants had predicted resistance detected only by NGS (including 3 participants with predicted resistance to two drugs); one participant had resistance for TDF (NGS threshold, 1%), eight for ETR (1% to 15%), and five for RPV (1% to 10%). Resistance predicted by Sanger was not detected in 3 participants by NGS^20^ (for TDF, ETR, and RPV) and in one participant by NGS^15^ (for TDF).

**TABLE 4:** Participants with predicted low-intermediate-high drug resistance to potential future treatment options

Resistance predicted by:	No. of participants	Results (*n* [%])^*a*^ by ARV drug		
		TDF (*n* = 42)	ETR (*n* = 88)	RPV (*n* = 106)
NGS and Sanger	110	42^*b*^ (32)	88^*c*^ (67)	106^*c*^ (80)
NGS only	11	1^*d*^ (1)	8^*e*^ (6)	5^*f*^ (4)
Sanger only	3	2^*g*^ (2)	1^*h*^ (1)	1^*h*^ (1)

a The denominator for all proportions was the total number of study participants, *n* = 132.

b For one participant, at all thresholds except NGS^15^ and for two participants except NGS^20^.

c For one participant, at all thresholds except NGS^20^.

d For one participant, only at NGS^01^.

e For one participant, at all thresholds except NGS^02^, for two participants except NGS^05^, for five participants except NGS^10^, for seven participants except NGS^15^, and for eight participants except NGS^20^.

f For two participants at all thresholds except NGS^10^, for five participants except NGS^15^ for five participants except NGS^20^.

g For one participant at all thresholds except NGS^15^ and for two except NGS^20^.

h For one participant at all thresholds except NGS^20^.

**FIG 6:**
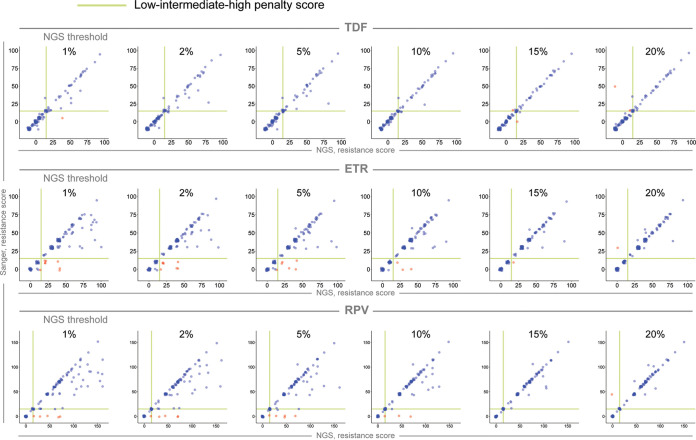
Predicted resistance scores to future treatment options for Sanger versus NGS. Scatterplots delineate the Sanger (*y* axes) and the six NGS thresholds (*x* axes) predicted resistance scores to TDF (top; range, 0 to 100), ETR (middle; range, 0 to 100), and RPV (bottom; range, 0 to 150). The boundaries of susceptible-potential low versus low-intermediate-high predicted resistance scores are highlighted by the green lines within each graph. Agreements in estimating resistance levels between technologies are shown by blue dots, and discrepancies are shown by red dots.

Scatterplots using actual resistance scores to future treatment options demonstrated overall good correlations between technologies in higher NGS thresholds, which was evident from the regression curves and tight confidence intervals, with gradual higher discrepancy toward lower thresholds (Fig. 6, note differences in scales).

### Sanger sequencing-compatible NGS threshold.

**FIG 7:**
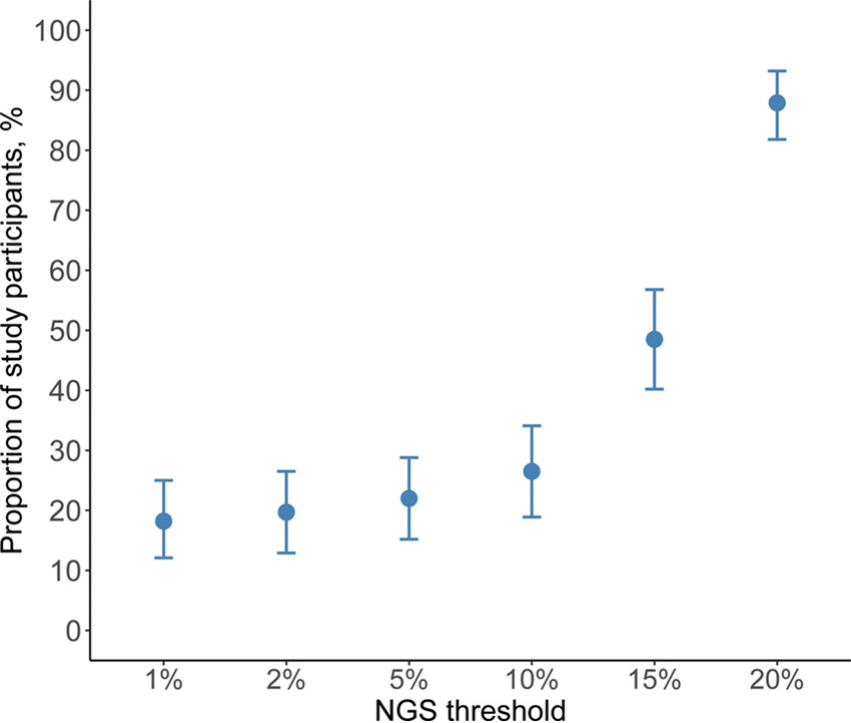
Distribution of study participants according to highest sequence similarly to Sanger by NGS threshold. The plot presents the percentage of participants (*y* axis) and their 95% bootstrapped confidence intervals that had their highest sequence similarity between Sanger and each of the six examined NGS thresholds (*x* axis).

In the fifth analysis, the average maximum percent similarity between technologies was 99.4% (median, 99.46; IQR, 99.16 to 99.7; range, 97.08% to 100%). Of the 132 participants, 79 (60%) had a unique optimal threshold, 19 (14%) had 2, 6 (5%) had 3, 3 (2%) had 4 or 5, and 22 (17%) participants had the same maximum similarity observed at all 6 NGS thresholds. In all 5,000 bootstrap samples, the 20% threshold had the highest similarity proportion. In addition, the bootstrapped confidence intervals confirmed the NGS threshold of 20% resulted in the highest maximum similarity. The distribution of the thresholds is demonstrated in Fig. 7 and shows that thresholds 1%, 2%, 5%, 10%, 15%, and 20% result in maximum similarity for 18.2%, 19.7%, 22.0%, 26.5%, 48.5%, and 87.9% of participants (NGS-Sanger pairs), respectively. Among the six examined NGS thresholds, the median percent nucleotide identity between Sanger sequencing and NGS was highest at 20% (99.5%; range, 97.1% to 100%), compared with 15% (99.4%; range, 95.4% to 100%), 10% (99.3%; range, 94.2% to 100%), 5% (98.9%; range, 93.7% to 100%), 2% (98.5%; range, 92.7% to 100%), or 1% (98.2%; range, 92.7% to 100%).

## DISCUSSION

In this study, we intended to identify the potential added value of next-generation over conventional Sanger sequencing for HIV-1 drug resistance testing, in 132 virologically unsuppressed and treatment-experienced Kenyan children and adolescents with diverse HIV-1 subtypes. Under the hypothesis that NGS is more sensitive than Sanger in DRM detection even in this cohort, with existing extensive resistance, we found that 12% to 38% of participants (depending on NGS threshold) had NRTI and NNRTI drug resistance profiles that differed between the two technologies, with elevated discrepancy at lower NGS thresholds, and that in 12% of participants, mutations detected only by NGS predicted higher ART resistance levels. These findings agree with the current knowledge of improved sensitivity of NGS over Sanger for DRM detection and extend them to a vulnerable population with already high resistance levels and with diverse HIV-1 subtypes, suggesting that upon further research, using NGS may potentially improve patient care.

The observed discrepancies in DRM detection between the two technologies, which should be expected (43), can essentially be considered minimal. However, we note that analyzed samples from this Kenyan youth cohort were obtained in 2010 to 2013, when HIV viral load monitoring was not implemented routinely, delaying treatment failure diagnosis. As a result, at sampling, individuals already had high levels of drug resistance, likely resulting in a weakened ability to detect an added value of the advanced DRM detection NGS technology (42). Therefore, the finding that even in this cohort NGS detected additional DRMs should support its potential added value over Sanger sequencing. However, we did not evaluate the longitudinal impact of these findings, and whether they provide additional clinically relevant information remains a critical question. Such relevance might be low if the identified low-frequency DRMs disappear over time, but they might be high if they evolve into major viral variants and impact drug susceptibility. A better understanding of mechanisms driving such decay or evolution could help resolve this important question.

Examination of the predicted reduced susceptibilities to future ART potentially caused by the minority resistance variants suggested a potential limitation of future regimens. These concerns could be alleviated by increased availability to newer medications and ART classes with high genetic barrier to resistance (42). However, the capacity to better detect potentially important low level DRMs must continue to be considered, and algorithms allowing reliable predictions of their relevance should continue to be developed, as drug resistance testing access is expanded globally for care, surveillance, or research purposes (3).

Better agreement in nucleotide identity between the examined sequencing technologies was observed at higher (15% to 20%) rather than lower NGS thresholds, with the highest agreement seen at 20%, although the range was overall high for all thresholds. These data support previous reports on laboratory isolates (44); extend them to real life, multisubtype data; and reinforce the practice that at higher NGS thresholds, Sanger sequencing and NGS can practically be used interchangeably. A better understanding of the discrepancy in detection of minority resistance variants at lower NGS levels is needed, as more global laboratories and settings consider adopting NGS for drug resistance testing and as the phenotypic and clinical impact of these minority resistance variants are being characterized (45, 46). Additional important considerations in this regard are higher costs, training, technical maintenance, and continuous bioinformatics support that might be associated with NGS technology.

The vast majority of the discrepancy in DRM detection between the two technologies was, as expected, due to mutation detection by NGS but not by Sanger. Negligible proportions of DRMs were identified by Sanger and not at high (15% to 20%) NGS thresholds, likely due to the stochastic nature of PCR amplification. Further examination of the differences in DRM detection between the two examined technologies demonstrated that the extent of agreement did not differ by amino acid position but that more prevalent DRMs had lower agreement. The etiology of these observations is hard to ascertain. They may be related to low numbers, sampling, transient and compensatory mutations, or specific drug selection pressure, as well as alternative reasons. Examination of the significance of these findings should continue as we evaluate advanced technologies in HIV drug resistance testing.

This study has several limitations. First, as discussed above, this cohort of Kenyan children and adolescents was already saturated with DRMs, making the specific NGS added value evaluation in this population more challenging. Second, amplicons for Sanger and NGS originated from different PCR amplifications, which could account for at least some of the observed differences between the technologies. Due to the stochastic nature of PCR amplification, this limitation could impact the performed discrepancy comparisons between Sanger and NGS, particularly at low NGS thresholds, and should be explored further. Third, the frequencies of minority resistance variants could be inaccurate, as we did not incorporate technology such as primer ID (47–50). Fourth, samples from participants were obtained about a decade ago, and some regimens are already outdated. Nevertheless, the addressed concepts remain relevant for current ART. Lastly, data presented here require phenotypic and clinical validation to confirm the potential benefit of the suggested added value of NGS and its potential impact on clinical care, resistance surveillance, and public health, which is work that is an ongoing research endeavor.

In summary, our findings suggest a potential added value of NGS over existing, less sensitive technology in detecting potentially important HIV-1 DRMs. The uniqueness of this Kenyan youth cohort with diverse HIV-1 subtypes, which already has high levels of drug resistance, likely weakening the ability to demonstrate such added value, further highlights the potential significance of these findings and the need for further research to corroborate them. The good agreement between the two sequencing technologies at high NGS thresholds continues to support their interchangeable use that is already occurring. However, the existing discrepancies mostly in lower thresholds and the significance of mutations identified at these low thresholds to patient care should continue to be explored.

## MATERIALS AND METHODS

### Study participants.

Children and adolescents were enrolled at the Academic Model Providing Access to Healthcare (AMPATH), a large HIV care program in western Kenya (51, 52), if they were perinatally HIV infected, receiving or initiating NNRTI-based 1st-line ART, and enrolled in a prior study during 2010 to 2013. Evaluation of ART adherence, viral suppression, drug resistance, and treatment outcomes in this cohort have been reported previously (42, 53, 54). In the current study, participants with detectable viral loads were included if they had both Sanger and NGS sequences performed on a blood plasma sample.

### HIV-1 sequencing.

HIV-1 RNA was extracted from blood plasma and was used as a template for two-round amplification. For RNA extraction, 400 to 2,000 μL plasma was processed using the bioMérieux MiniMAG (Durham, NC) or EZ1 DSP virus kit (Qiagen, Germantown, MD). HIV-1 RNA was converted to cDNA by a SuperScript III first-strand synthesis system (Thermo-Fisher Scientific, Waltham, MA) and amplified in two rounds of PCR using Phusion high-fidelity DNA polymerase (New England BioLabs, Ipswitch, MA) and Platinum *Taq* DNA polymerase high fidelity (Thermo-Fisher Scientific) in the 1st and 2nd round, respectively.

Generated sequences encompassed the HIV-1 region encoding the first 240 codons of reverse transcriptase (HXB2 nucleotide positions 2550 to 3268). Sequencing was performed at the University of Rhode Island Genomics and Sequencing Center (https://web.uri.edu/gsc/sequencing-service/). For Sanger sequencing, the Applied Biosystems BigDye Terminator v3.1 chemistry and ABI 3130XL capillary electrophoresis analyzer were used, sequences were assembled with Sequencher v4.10.1 (Gene Codes Co., Ann Arbor, MI), and quality control was performed with SQUAT (55). For NGS, library preparation was performed using the Nextera DNA (Flex) library prep kit (Illumina) followed by sequencing on the Illumina MiSeq platform. Sequences were processed by the bioinformatics pipeline HIVMMER (56).

### HIV-1 subtyping.

HIV-1 subtyping was performed by using REGA (57) and COMET (58). Minor discrepancies were resolved on a case-by-case basis with manual inspection of sequence alignments, identifying recombination breakpoints (if any) and inferring phylogeny by RAxML v.8.10 (59).

### Drug resistance mutations.

NRTI- and NNRTI-associated DRMs were obtained from the Stanford Database mutation score tables (v8.9) (60, 61). For Sanger sequences, codons with one or two nucleotide International Union of Pure and Applied Chemistry (IUPAC) ambiguities were translated to all possible encoded amino acids and were included in the analyses. Codons with three nucleotide IUPAC ambiguities were excluded due to the unrealistic number of potentially encoded amino acids. For NGS data, DRMs were evaluated using amino acid frequency tables generated by the codon-aware bioinformatics pipeline HIVMMER (56). The following six NGS thresholds were used for the identification of DRMs: 1%, 2%, 5%, 10%, 15%, and 20%. At drug resistance positions with more than one amino acid mutation associated with resistance, each amino acid was examined separately.

### Sanger versus NGS comparison.

To examine agreement and discrepancy in identifying DRMs by the two sequencing technologies and their potential added value for clinical care, we performed the following five analyses:

(i) Any discrepancy of drug resistance. This analysis was an estimation of proportions of participants with identical and nonidentical DRMs identified by both technologies. Drug resistance profiles were considered identical if the complete DRM profiles (combined NRTI/NNRTI and within each drug class) were identified by both technologies. For this analysis, the total number of participants was used as the denominator. Participants with both DRMs identified only by Sanger and DRMs identified only by NGS were captured in both proportions and were considered nonidentical.
(ii) Specific DRMs. This analysis was an evaluation of the proportion and mean numbers of NRTI and NNRTI DRMs per participant that were identified by the two technologies.
(iii) Variation among specific DRMs. This analysis was an examination of agreement and discordance between DRM detection by Sanger and NGS at the 1% threshold. Specifically, we looked at the probability of DRM detection by NGS among those not detected by Sanger and assessed whether this probability differed across NRTI and NNRTI DRMs. We used likelihood ratio tests from fitted logistic regression models to assess whether discordance differs by DRM. To examine the relationship between DRM prevalence as measured by Sanger and probability of discordance between the sequencing technologies, we calculated the Spearman rank correlation between these discordance rates and the prevalence of specific DRMs by Sanger sequencing.
(iv) DRM impact on current and future treatment options. This analysis was an assessment of the impact of discordant DRMs between the two technologies on current and potential future reverse transcriptase inhibitor treatment options. Current ART regimens, used at the time of sampling, included lamivudine (3TC), abacavir (ABC), stavudine (D4T), zidovudine (AZT), efavirenz (EFV), and nevirapine (NVP). Potential future ARTs at that time that were used include tenofovir (TDF), etravirine (ETR), and rilpivirine (RPV). Agreement and discrepancy among technologies were determined using Stanford Database resistance prediction tools. Discrepancy was defined as a DRM detected by one technology and not the other that led to a potentially clinically relevant change in predicted resistance to a drug, from susceptible or potential-low-level (levels 1 and 2) to a low-intermediate-high-level (levels 3 to 5) resistance. As in the first analysis above, the technologies were considered identical only if predictions for all drugs were identical. In addition, to allow more sensitivity exploring discrepancies, we then used the actual Stanford Database penalty scores (rather than the predicted resistance levels used above) to compare the technologies’ prediction of resistance to future treatment options. We used scatterplots to visualize relationships between Sanger and NGS, and in order to link penalty scores with resistance levels, we highlighted score boundaries associated with susceptible-potential-low vs. low-intermediate-high predicted resistance levels within each scatterplot.
(v) Sanger-compatible NGS threshold. This analysis included the determination of the Sanger-compatible NGS threshold using the estimated nucleotide identity between consensus sequences generated by Sanger sequencing and each NGS threshold. Pairwise TN93 distances were computed by using the tn93 package (https://github.com/veg/tn93) and averaging distances at ambiguous nucleotide positions. To find the optimal NGS threshold among the 6 examined here (1%, 2%, 5%, 10%, 15%, and 20%), we captured the NGS threshold(s) for each participant that resulted in the maximum observed NGS/Sanger similarity. For some participants, the same maximum similarity occurred at more than one threshold. For each threshold, we captured the percentage of participants who had their observed maximum similarity observed at that threshold. Bootstrap was used to calculate 95% confidence intervals for the thresholds, using 5,000 runs and sampling each participant with replacement.

All statistical analyses were performed in R v.3.6.0 (62).
