# Supplementary material for: Added Value of Next Generation over Sanger Sequencing in Kenyan Youth with Extensive HIV-1 Drug Resistance
Source: Microbiol Spectr. 2022 Nov 29;10(6):e03454-22. doi: 10.1128/spectrum.03454-22 (PMC9769539; doi:10.1128/spectrum.03454-22)
Supplement: Supplemental file 1 — Tables S1 and S2. Download spectrum.03454-22-s0001.pdf, PDF file, 0.1 MB [file spectrum.03454-22-s0001.pdf]

Supplementary Table S1. Discrepancy in detecting specific NRTI DRMs

| DRM | Proportion detected by Sanger | Proportion detected by NGS among those not detected by Sanger, P(N(k) = 1 \| S = 0) | | | | | | Proportion not detected by Sanger among those detected by NGS, P(S = 0 \| N(k) = 1) | | | | | |
|---|---|---|---|---|---|---|---|---|---|---|---|---|---|
| | | *NGS threshold* | | | | | | *NGS threshold* | | | | | |
| | | 1% | 2% | 5% | 10% | 15% | 20% | 1% | 2% | 5% | 10% | 15% | 20% |
| **M41L** | 12.9% (17/132) | 0.9% (1/115) | 0% (0/115) | 0% (0/115) | 0% (0/115) | 0% (0/115) | 0% (0/115) | 5.9% (1/17) | 0% (0/16) | 0% (0/16) | 0% (0/16) | 0% (0/16) | 0% (0/16) |
| **A62V** | 0.8% (1/132) | 0% (0/131) | 0% (0/131) | 0% (0/131) | 0% (0/131) | 0% (0/131) | 0% (0/131) | 0% (0/1) | 0% (0/1) | 0% (0/1) | 0% (0/1) | 0% (0/1) | 0% (0/1) |
| **K65R** | 9.1% (12/132) | 0% (0/120) | 0% (0/120) | 0% (0/120) | 0% (0/120) | 0% (0/120) | 0% (0/120) | 0% (0/12) | 0% (0/12) | 0% (0/12) | 0% (0/12) | 0% (0/12) | 0% (0/11) |
| **D67NTEG** | 14.4% (19/132) | 1.8% (2/113) | 1.8% (2/113) | 1.8% (2/113) | 0.9% (1/113) | 0% (0/113) | 0% (0/113) | 9.5% (2/21) | 9.5% (2/21) | 9.5% (2/21) | 5% (1/20) | 0% (0/19) | 0% (0/17) |
| **T69D** | 1.5% (2/132) | 0% (0/130) | 0% (0/130) | 0% (0/130) | 0% (0/130) | 0% (0/130) | 0% (0/130) | 0% (0/2) | 0% (0/2) | 0% (0/2) | 0% (0/2) | 0% (0/2) | 0% (0/2) |
| **K70RNQ** | 18.2% (24/132) | 2.8% (3/108) | 1.9% (2/108) | 1.9% (2/108) | 1.9% (2/108) | 0.9% (1/108) | 0.9% (1/108) | 11.1% (3/27) | 7.7% (2/26) | 7.7% (2/26) | 7.7% (2/26) | 4% (1/25) | 4.3% (1/23) |
| **L74VI** | 34.1% (45/132) | 5.7% (5/87) | 5.7% (5/87) | 4.6% (4/87) | 3.4% (3/87) | 1.1% (1/87) | 1.1% (1/87) | 10% (5/50) | 10% (5/50) | 8.2% (4/49) | 6.2% (3/48) | 2.2% (1/46) | 2.2% (1/46) |
| **V75MIT** | 8.3% (11/132) | 0.8% (1/121) | 0.8% (1/121) | 0.8% (1/121) | 0% (0/121) | 0% (0/121) | 0% (0/121) | 8.3% (1/12) | 8.3% (1/12) | 8.3% (1/12) | 0% (0/11) | 0% (0/11) | 0% (0/11) |
| **Y115F** | 27.3% (36/132) | 4.2% (4/96) | 4.2% (4/96) | 4.2% (4/96) | 1% (1/96) | 1% (1/96) | 0% (0/96) | 10% (4/40) | 10% (4/40) | 10% (4/40) | 2.7% (1/37) | 2.7% (1/37) | 0% (0/36) |
| **F116Y** | 1.5% (2/132) | 0% (0/130) | 0% (0/130) | 0% (0/130) | 0% (0/130) | 0% (0/130) | 0% (0/130) | 0% (0/2) | 0% (0/2) | 0% (0/2) | 0% (0/2) | 0% (0/2) | 0% (0/2) |
| **Q151M** | 0.8% (1/132) | 0% (0/131) | 0% (0/131) | 0% (0/131) | 0% (0/131) | 0% (0/131) | 0% (0/131) | 0% (0/1) | 0% (0/1) | 0% (0/1) | 0% (0/1) | 0% (0/1) | 0% (0/1) |
| **M184VI** | 88.6% (117/132) | 6.7% (1/15) | 6.7% (1/15) | 6.7% (1/15) | 6.7% (1/15) | 0% (0/15) | 0% (0/15) | 0.8% (1/118) | 0.8% (1/118) | 0.8% (1/118) | 0.8% (1/118) | 0% (0/117) | 0% (0/117) |
| **L210W** | 4.5% (6/132) | 1.6% (2/126) | 1.6% (2/126) | 1.6% (2/126) | 0.8% (1/126) | 0% (0/126) | 0% (0/126) | 25% (2/8) | 25% (2/8) | 25% (2/8) | 14.3% (1/7) | 0% (0/6) | 0% (0/6) |
| **T215FVIYAL** | 22.7% (30/132) | 3.9% (4/102) | 2.9% (3/102) | 1% (1/102) | 1% (1/102) | 1% (1/102) | 1% (1/102) | 11.8% (4/34) | 9.1% (3/33) | 3.2% (1/31) | 3.2% (1/31) | 3.3% (1/30) | 3.3% (1/30) |

| | | | | | | | | | | | | | |
|---|---|---|---|---|---|---|---|---|---|---|---|---|---|
| **K219EQN** | 17.4% (23/132) | 0.9% (1/109) | 0.9% (1/109) | 0% (0/109) | 0% (0/109) | 0% (0/109) | 0% (0/109) | 4.2% (1/24) | 4.2% (1/24) | 0% (0/23) | 0% (0/23) | 0% (0/23) | 0% (0/22) |

Footnote: Abbreviations: DRMs, drug resistant mutations; NGS, next generation sequencing; NRTI, nucleoside reverse transcriptase inhibitors; NNRTI, non-nucleoside reverse transcriptase inhibitors.

Supplementary Table S2. Discrepancy in detecting specific NNRTI DRMs

| DRM | Proportion detected by Sanger | Proportion detected by NGS among those not detected by Sanger, P(N(k) = 1 \| S = 0) | | | | | | Proportion not detected by Sanger among those detected by NGS, P(S = 0 \| N(k) = 1) | | | | | |
|---|---|---|---|---|---|---|---|---|---|---|---|---|---|
| | | *NGS threshold* | | | | | | *NGS threshold* | | | | | |
| | | 1% | 2% | 5% | 10% | 15% | 20% | 1% | 2% | 5% | 10% | 15% | 20% |
| **A98G** | 8.3% (11/132) | 3.3% (4/121) | 3.3% (4/121) | 3.3% (4/121) | 1.7% (2/121) | 0.8% (1/121) | 0% (0/121) | 26.7% (4/15) | 26.7% (4/15) | 26.7% (4/15) | 15.4% (2/13) | 8.3% (1/12) | 0% (0/11) |
| **L100I** | 1.5% (2/132) | 0.8% (1/130) | 0% (0/130) | 0% (0/130) | 0% (0/130) | 0% (0/130) | 0% (0/130) | 33.3% (1/3) | 0% (0/2) | 0% (0/2) | 0% (0/2) | 0% (0/2) | 0% (0/2) |
| **K101EPH** | 13.6% (18/132) | 3.5% (4/114) | 1.8% (2/114) | 0.9% (1/114) | 0% (0/114) | 0% (0/114) | 0% (0/114) | 18.2% (4/22) | 10% (2/20) | 5.3% (1/19) | 0% (0/18) | 0% (0/18) | 0% (0/18) |
| **K103NS** | 37.1% (49/132) | 2.4% (2/83) | 2.4% (2/83) | 2.4% (2/83) | 1.2% (1/83) | 1.2% (1/83) | 0% (0/83) | 3.9% (2/51) | 3.9% (2/51) | 3.9% (2/51) | 2% (1/50) | 2% (1/50) | 0% (0/49) |
| **V106IMA** | 8.3% (11/132) | 0.8% (1/121) | 0.8% (1/121) | 0.8% (1/121) | 0.8% (1/121) | 0.8% (1/121) | 0% (0/121) | 8.3% (1/12) | 8.3% (1/12) | 8.3% (1/12) | 8.3% (1/12) | 8.3% (1/12) | 0% (0/11) |
| **V108I** | 8.3% (11/132) | 3.3% (4/121) | 2.5% (3/121) | 0% (0/121) | 0% (0/121) | 0% (0/121) | 0% (0/121) | 26.7% (4/15) | 21.4% (3/14) | 0% (0/11) | 0% (0/11) | 0% (0/11) | 0% (0/11) |
| **E138QKAG** | 12.1% (16/132) | 2.6% (3/116) | 2.6% (3/116) | 1.7% (2/116) | 0% (0/116) | 0% (0/116) | 0% (0/116) | 15.8% (3/19) | 15.8% (3/19) | 11.1% (2/18) | 0% (0/16) | 0% (0/16) | 0% (0/16) |
| **V179ELD** | 2.3% (3/132) | 3.1% (4/129) | 2.3% (3/129) | 1.6% (2/129) | 0.8% (1/129) | 0.8% (1/129) | 0.8% (1/129) | 57.1% (4/7) | 50% (3/6) | 40% (2/5) | 25% (1/4) | 25% (1/4) | 33.3% (1/3) |
| **Y181CVI** | 50.8% (67/132) | 7.7% (5/65) | 6.2% (4/65) | 6.2% (4/65) | 6.2% (4/65) | 1.5% (1/65) | 0% (0/65) | 6.9% (5/72) | 5.6% (4/71) | 5.7% (4/70) | 5.7% (4/70) | 1.5% (1/67) | 0% (0/65) |
| **Y188LCHF** | 3% (4/132) | 2.3% (3/128) | 1.6% (2/128) | 0% (0/128) | 0% (0/128) | 0% (0/128) | 0% (0/128) | 42.9% (3/7) | 33.3% (2/6) | 0% (0/4) | 0% (0/4) | 0% (0/4) | 0% (0/4) |

| | | | | | | | | | | | | | |
|---|---|---|---|---|---|---|---|---|---|---|---|---|---|
| **G190AES** | 26.5% (35/132) | 3.1% (3/97) | 1% (1/97) | 1% (1/97) | 1% (1/97) | 0% (0/97) | 0% (0/97) | 7.9% (3/38) | 2.8% (1/36) | 2.8% (1/36) | 2.8% (1/36) | 0% (0/35) | 0% (0/35) |
| **H221Y** | 24.2% (32/132) | 8% (8/100) | 7% (7/100) | 6% (6/100) | 4% (4/100) | 1% (1/100) | 1% (1/100) | 20% (8/40) | 17.9% (7/39) | 15.8% (6/38) | 11.1% (4/36) | 3% (1/33) | 3.2% (1/31) |
| **P225H** | 1.5% (2/132) | 0.8% (1/130) | 0.8% (1/130) | 0% (0/130) | 0% (0/130) | 0% (0/130) | 0% (0/130) | 33.3% (1/3) | 33.3% (1/3) | 0% (0/2) | 0% (0/2) | 0% (0/2) | 0% (0/2) |
| **F227L** | 2.3% (3/132) | 1.6% (2/129) | 0.8% (1/129) | 0% (0/129) | 0% (0/129) | 0% (0/129) | 0% (0/129) | 40% (2/5) | 25% (1/4) | 0% (0/3) | 0% (0/3) | 0% (0/3) | 0% (0/3) |
| **M230LI** | 2.3% (3/132) | 1.6% (2/129) | 1.6% (2/129) | 1.6% (2/129) | 0.8% (1/129) | 0% (0/129) | 0% (0/129) | 40% (2/5) | 40% (2/5) | 40% (2/5) | 25% (1/4) | 0% (0/3) | 0% (0/3) |
| **K238NT** | 4.5% (6/132) | 0.8% (1/126) | 0.8% (1/126) | 0.8% (1/126) | 0% (0/126) | 0% (0/126) | 0% (0/126) | 14.3% (1/7) | 14.3% (1/7) | 14.3% (1/7) | 0% (0/6) | 0% (0/6) | 0% (0/6) |

Footnote: Abbreviations: DRMs, drug resistant mutations; NGS, next generation sequencing; NRTI, nucleoside reverse transcriptase inhibitors; NNRTI, non-nucleoside reverse transcriptase inhibitors.
